# Risk of Pneumonia is associated with Antipsychotic Drug Use among older patients with Parkinson's Disease: A Case-control Study

**DOI:** 10.7150/ijms.63246

**Published:** 2021-08-26

**Authors:** Kuang-Hua Huang, Wei-Yin Kuo, Yu-Hsiang Kuan, Yu-Chia Chang, Tung-Han Tsai, Chien-Ying Lee

**Affiliations:** 1 Department of Health Services Administration, China Medical University, Taichung, Taiwan.; 2 Department of Pharmacology, Chung Shan Medical University, Taichung, Taiwan.; 3 Department of Pharmacy, Chung Shan Medical University Hospital, Taichung, Taiwan.; 4 Department of Healthcare Administration, Asia University, Taichung, Taiwan.; 5 Department of Long Term Care, National Quemoy University, Kinmen, Taiwan.

**Keywords:** pneumonia, antipsychotics, Parkinson's disease, pharmacoepidemiology

## Abstract

**Objective:** To investigate the risk of pneumonia associated with the use of antipsychotic drugs in older-adult patients with Parkinson's disease (PD) in Taiwan.

**Methods:** This case-control study was based on data from the longitudinal health insurance database in Taiwan. We analyzed the data of 51,158 older patients with PD for the period between 2001 and 2016. To reduce the potential confounding caused by unbalanced covariates in nonexperimental settings, we used propensity score matching to include older patients without pneumonia to serve as the control group.

**Results:** Compared with patients who had never taken antipsychotics, current (adjusted odds ratios [aOR] =1.63, 95% confidence interval [CI] = 1.51-1.75), recent (aOR = 1.63, 95% CI = 1.52-1.74), and past (aOR = 1.89, 95% CI = 1.80-2.00) users of antipsychotics had a higher risk of incident pneumonia. Among typical and atypical antipsychotics, haloperidol and clozapine were associated with higher risks of incident pneumonia, respectively. By contrast, aripiprazole was not associated with a higher risk of pneumonia.

**Conclusion:** Older patients with PD receiving typical antipsychotics or atypical antipsychotics had a higher risk of pneumonia. Among these antipsychotics, clozapine had the highest risk of pneumonia. Clinicians should pay attention to the risk of pneumonia in older patients with PD who receive typical antipsychotics and atypical antipsychotics.

## Introduction

Parkinson's disease (PD) is the second most common age-related motor neurodegenerative disorder first described in the early 1800s by James Parkinson 1. As PD progresses, the bulbar muscles are affected, leading to dysphagia. Dysphagia is a common symptom in patients with PD and may occur at any stage in the disease course. Most patients with PD develop oropharyngeal dysphagia in the early stage 2. Oropharyngeal dysphagia is the sensation of difficulty or an abnormal delay in the movement of a food bolus from the oropharynx to the stomach. Those with PD often report difficulty swallowing, which is also associated with a high risk of aspiration pneumonia 3. A 10-fold higher incidence of aspiration pneumonia was observed in patients with PD compared with patients without PD 4. A meta-analysis revealed that the risk of oropharyngeal dysphagia in individuals with PD was approximately three times greater than that in healthy older adults 5. Aspiration pneumonia is the leading cause of death in patients with PD, and it is estimated to account for 70% of the mortality in this group 6.

More than half of all patients with PD eventually develop PD psychosis, typically after 10 or more years of treatment 7. The prevalence of various psychiatric disorders is high among those with PD. Antipsychotic drugs are often used for the treatment of behavioral and psychological symptoms in patients with PD 8. Several observational studies have explored the relationship between the use of antipsychotic drugs and the risk of community-acquired pneumonia, mainly in older patients 9-11. A systematic review and meta-analysis suggested that exposure to typical and atypical antipsychotic drugs is associated with a significantly increased risk of pneumonia in all age groups 12. Only one study indicated that the risk of pneumonia was significantly higher for patients with PD who used unsuitable second-generation antipsychotic drugs compared with those taking the appropriate drugs, according to the American Geriatrics Society (AGS) 2015 Beers criteria in older patients 13.

However, few studies have examined the risk of pneumonia associated with the use of antipsychotic medications among patients with PD. Because antipsychotic agents are associated with an increased risk of pneumonia, we hypothesized that patients with PD have a higher risk of all-cause pneumonia. Understanding the risk of pneumonia in patients with PD is critical for clinicians. Therefore, we examined the risk of pneumonia associated with the use of antipsychotic drugs in older patients with PD and investigated the related risk factors for pneumonia by using nationwide data from Taiwan's longitudinal health insurance database (LHID).

## Methods

### Database

This study was a case-control study, in which secondary data analysis was conducted. Data were obtained from the LHID released by the Health and Welfare Data Science Center, Ministry of Health and Welfare Taiwan (HWDC, MOHW). The LHID comprises the information of two million beneficiaries randomly selected from the Taiwan National Health Insurance (NHI) program. The NHI program is a nationwide social insurance program that has covered up to 99% of citizens since 1995. Hence, the database is nationally representative of Taiwan. Owing to the anonymity of the database, the requirement for informed consent was waived, and this study was approved as an ethical review by the Institutional Review Board of China Medical University Hospital, Taiwan (No. CMUH107-REC2-004).

### Study participants

All study participants were older patients (aged ≥65 years) with PD (International Classification of Diseases, Ninth Revision, Clinical Modification [ICD-9-CM]: 332; International Classification of Diseases, Tenth Revision, Clinical Modification [ICD-10-CM]: G20) at any period from 2001 to 2016. Older patients who received a principal diagnosis of pneumonia (ICD-9-CM: 480-486, ICD-10-CM: J12-J18) comprised the case group. To reduce the potential confounding caused by unbalanced covariates in nonexperimental settings, we used 1:4 propensity score matching to include older patients without pneumonia to serve as the control group. The propensity score of the study was the probability of patients incident pneumonia, calculated by sex, age, income level, urbanization, and Charlson comorbidity index (CCI).

### Study design

In this case-control study, we investigated the risk of pneumonia associated with incident antipsychotic use among older patients with PD. We defined antipsychotic use by the following Anatomic Therapeutic Chemical classification system codes: typical antipsychotics-haloperidol (N05AD01) and chlorpromazine (N05AA01)-and atypical antipsychotics-clozapine (N05AH02), olanzapine (N05AH03), quetiapine (N05AH04), aripiprazole (N05AX12), and risperidone (N05AX08). The observation period for assessing the antipsychotic use of each patient was the full year before the pneumonia diagnosis. The study calculated the duration of receiving antipsychotic drugs for each study subject. The definition of “current use” was the medication duration covered the date of incident pneumonia or ended at most 30 days before pneumonia; Antipsychotic use was categorized as ''recent use'' if the medication duration ended 31-90 days before pneumonia; If the medication duration ended more than 90 days was defined as "past use". Patients who had never been prescribed an antipsychotic before their pneumonia diagnosis served as the reference group.

### Statistical analysis

We investigated the association between antipsychotic drugs and pneumonia through a conditional logistic regression. Each antipsychotic drug was seen as an independent variable. The study subjects may receive two or more kinds of antipsychotic drugs in the observation period. The conditional logistic regression analysis would estimate the odds ratio of each antipsychotic by adjusted all independent variables. The control variables were sex, age, income level, urbanization, CCI score, and comorbidities related to pneumonia. The following comorbidities were considered: diabetes mellitus (ICD-9-CM: 250; ICD-10-CM: E08-E13), hypertension (ICD-9-CM: 401-405; ICD-10-CM: I10-I13, I15), cerebrovascular disease (ICD-9-CM: 430-438; ICD-10-CM: I60-I69), arrhythmia (ICD-9-CM: 427; ICD-10-CM: I47-I49), upper respiratory tract infection (ICD-9-CM: 465.9; ICD-10-CM: J00-06, J30-39), heart failure (ICD-9-CM: 428.0; ICD-10-CM: I50), asthma (ICD-9-CM: 493, ICD-10-CM: J45), chronic obstructive pulmonary disease (COPD; ICD-9-CM: 490-492, 494-496; ICD-10-CM: J40-J44, J47), periodontitis (ICD-9-CM: 523; ICD-10-CM: K05.4), chronic kidney disease (ICD-9-CM: 585; ICD-10-CM: N18), liver disease (ICD-9-CM: 571; ICD-10-CM: K70-K76), alcoholism (ICD-9-CM: 303, ICD-10-CM: F10.2), Alzheimer's disease (ICD-9-CM: 331.0, 290.1; ICD-10-CM: G30, F00), rheumatoid arthritis (ICD-9-CM: 714; ICD-10-CM: M05-M06, M45), cancer (ICD-9-CM: 140-239; ICD-10-CM: C00-C97), and epilepsy (ICD-9-CM: 345; ICD-10-CM: G40-G41). SAS version 9.4 (SAS Institute Inc., Cary, NC, USA) was used for statistical analysis, and statistical significance was indicated if p < 0.05.

## Results

### Baseline characteristics of the sample

Table 1 presents the baseline characteristics of the sample. After matching was conducted, the data of 51,158 older patients with PD were included for analysis, including 10,237 and 40,948 patients with and without incident pneumonia, respectively. The mean age of the patients with pneumonia was 77.10 ± 5.84 years. As expected, the distributions of sex, age, income level, urbanization, and CCI did not significantly differ between the case and control groups after matching. In the case group, 34.59% of patients had diabetes mellitus, 68.67% had hypertension, 59.83% had cerebrovascular disease, 18.57% had arrhythmia, 51.32% had upper respiratory tract infection, 17.96% had heart failure, 21.96% had asthma, 57.99% had COPD, 2.51% had periodontitis, 2.51% had chronic kidney disease, 10.71% had chronic liver disease, 18.36% had Alzheimer disease, 2.51% had rheumatoid arthritis, 17.93% had cancer, 11.70% had epilepsy, and 6.96% had major depressive disorder.

### Incidence of pneumonia with antipsychotic use

Table 2 lists the incidence rate of pneumonia with antipsychotic use. The incidence rate of pneumonia was 19.06% in patients who had never received any antipsychotics, 30.49% in current users, 29.88% in recent users, and 31.39% in past users (p < 0.001). As for typical antipsychotic use, incident pneumonia was diagnosed in 19.84% of patients who had never taken any antipsychotics, in 35.23% of current users, in 31.17% of recent users, and in 28.69% of past users (p < 0.001). As for atypical antipsychotics use, incident pneumonia was diagnosed in 19.22% of patients who had never taken any antipsychotics, in 29.85% of current users, in 29.71% of recent users, and in 32.28% of past users (p < 0.001).

The distribution of incident pneumonia among the usage groups differed significantly for individual typical antipsychotics (p < 0.001). Incident pneumonia also differed significantly between each atypical antipsychotic, except for aripiprazole.

### Association of incident pneumonia and antipsychotic use

Table 3 reveals the adjusted odds ratios (aORs) for antipsychotics after sex, age, income level, urbanization, and related comorbidities were controlled for. Compared with patients who had never taken antipsychotics, those currently taking antipsychotics had a higher risk of incident pneumonia (aOR = 1.63, 95% confidence interval [CI] =1.51-1.75), recent users (aOR = 1.63, 95% CI = 1.52-1.74), and past users (aOR = 1.89, 95% CI = 1.80-2.00). As for typical antipsychotics, compared with patients who had never taken typical antipsychotics, incident pneumonia risk was higher in current users (aOR = 1.76, 95% CI = 1.46-2.13), recent users (aOR = 1.46, 95% CI = 1.23-1.73), and past users (aOR = 1.10, 95% CI = 1.00-1.21). As for atypical antipsychotics, compared with patients who had never taken atypical antipsychotics, incident pneumonia risk was higher in current users (aOR = 1.57, 95% CI = 1.45-1.70), recent users (aOR = 1.61, 95% CI = 1.50-1.73), and past users (aOR = 1.94, 95% CI = 1.83-2.05). Among typical antipsychotics, haloperidol had a higher risk of incident pneumonia for both current users (aOR = 1.72, 95% CI = 1.41-2.11) and recent users (aOR = 1.41, 95% CI = 1.17-1.69). For chlorpromazine, only current users had a higher risk (aOR = 1.84, 95% CI = 1.13-2.98).

Among atypical antipsychotics, clozapine had a higher risk of incident pneumonia regardless of whether its use was current (aOR = 1.77, 95% CI = 1.24-2.51), recent (aOR = 2.60, 95% CI = 1.95-3.46), or in the past (aOR = 1.81, 95% CI = 1.46-2.23). Patients with current (aOR = 1.60, 95% CI = 1.21-2.51) or past (aOR = 1.20, 95% CI = 1.03-1.40) olanzapine use had a higher risk of incident pneumonia, compared with those who had never taken atypical antipsychotics. Finally, compared with those who had never taken atypical antipsychotics, both quetiapine users (current: aOR = 1.44, 95% CI = 1.31-1.58; recent user: aOR = 1.53, 95% CI = 1.41-1.66; past users: aOR = 1.71, 95% CI = 1.60-1.82), and risperidone users (current user: aOR = 1.81, 95% CI = 1.55-2.11; recent user: aOR = 1.62, 95% CI = 1.41-1.86; past users: aOR = 1.52, 95% CI = 1.39-1.66) had a higher risk of incident pneumonia.

Patients with diabetes mellitus, hypertension, upper respiratory tract infection, chronic liver disease, rheumatoid arthritis, cancer, and anxiety had a lower risk of incident pneumonia, whereas patients with cerebrovascular disease, cerebrovascular disease, asthma, COPD, alcoholism, Alzheimer disease, epilepsy, schizophrenia, and bipolar disorder had a higher risk of incident pneumonia.

## Discussion

Oropharyngeal dysphagia is a frequent symptom in neurological disease. In neurodegenerative disorders such as PD and related disorders, as the disease progresses, patients have an increased risk of rapidly developing dysphagia 5. Patients with PD are at greater risk of aspiration pneumonia than are individuals in the general population 14. Because no studies have examined whether those with PD who use antipsychotic drugs also have a higher risk of pneumonia, we investigated the risk of pneumonia associated with the use of antipsychotic drugs among older patients with PD.

Antipsychotics have varying degrees of anticholinergic effects and could lead to aspiration pneumonia because of dry mouth and impaired oropharyngeal bolus transport 15. Anticholinergic drug use is a risk factor for pneumonia in older patients. A Taiwanese study indicated that older patients receiving anticholinergic drugs have an increased risk of incident pneumonia 16. One established mechanism for pneumonia is dry mouth, which is frequently caused by the anticholinergic side effects of medications; a dry mouth may lead to oropharyngeal swallowing impairment, which may result in aspiration pneumonia and even lead to death 17. Anticholinergic burden scores for drugs in Germany indicated that antipsychotics such as aripiprazole and risperidone have weak anticholinergic effects (score = 1); haloperidol, olanzapine, and quetiapine have moderate anticholinergic effects (score = 2); and clozapine has strong anticholinergic effects (score = 3) 18. Another scale for anticholinergic activity drugs used in Brazil indicated that antipsychotics such as aripiprazole, haloperidol, and risperidone have weak anticholinergic effects (score = 1); olanzapine and quetiapine have moderate anticholinergic effects (score = 2); and clozapine and chlorpromazine have strong anticholinergic effects (score = 3) 19. Based on these two scales, we included seven drugs, namely haloperidol, chlorpromazine, olanzapine, quetiapine, risperidone, clozapine, and aripiprazole, to investigate the relationship between antipsychotic drugs and pneumonia risk among older patients with PD. Our results indicate that older patients with PD who receive antipsychotics had a higher risk of pneumonia. Compared with patients who had never taken antipsychotics, current users, (aOR = 1.63, 95% CI = 1.51-1.75), recent users (aOR = 1.63, 95% CI = 1.52-1.74), and past users (aOR = 1.89, 95% CI = 1.80-2.00) had a higher risk of incident pneumonia.

Persistent psychotic symptoms may develop in up to 40%-60% of patients with PD, but estimates vary widely 7, 20, 21. Evidence suggests that people with psychiatric disorders are at an increased risk of common infectious diseases 22. For the treatment of patients with PD, the use of atypical antipsychotics (e.g., clozapine, pimavanserin, or quetiapine) should be prioritized; typical antipsychotics (e.g., haloperidol) are not recommended 23. A meta-analysis suggested that the risk of pneumonia was significantly increased by exposure to typical antipsychotic drugs (OR = 1.68, 95% CI = 1.39-2.04) and atypical antipsychotic drugs (OR = 1.98, 95% CI = 1.67-2.35) in all age groups 12. Our study also revealed that older patients with PD who receive typical antipsychotics and atypical antipsychotics had a higher risk of incident pneumonia.

Our results indicated that current, recent, or past use of clozapine leads to the highest risk of pneumonia of all the studied antipsychotic medications; this result may be attributed to clozapine's strong anticholinergic effect 18, 19. We also found that those taking aripiprazole, an atypical antipsychotic, did not have an elevated risk of pneumonia; this finding may be attributed to aripiprazole's weak anticholinergic effect 18, 19. The scale used in Brazil indicated that chlorpromazine has a strong anticholinergic effect 20, whereas data from Germany indicated that chlorpromazine has no anticholinergic effect 19. Thus, further investigation is required to clarify the causal relationship between chlorpromazine and pneumonia. Antipsychotics have immunoregulatory effects and anti-inflammatory effects, and clozapine may have inflammatory effects 24. Clozapine exposure may be associated with an increased risk of pneumonia when used individually and with an even higher risk when treatment is combined with other antipsychotics 25. Some atypical antipsychotics, such as clozapine, quetiapine, or risperidone, can modulate the cytokine network 26, 27, and clozapine directly influences the plasma levels of several cytokines that resemble an inflammatory reaction 24; clozapine might also enhance susceptibility to infections during treatment 28.

The main strength of our study is that it is the first to investigate the relationship between antipsychotic use and pneumonia risk in older patients with PD by dividing the follow-up period into current use, recent use, and past use. Compared with patients who had never taken typical antipsychotics, current (aOR = 1.76, 95% CI = 1.46-2.13), recent (aOR = 1.46, 95% CI = 1.23-1.73), and past (aOR = 1.10, 95% CI = 1.00-1.21) users of typical antipsychotics had a higher risk of incident pneumonia. Furthermore, compared with patients who had never taken atypical antipsychotics, current (aOR = 1.57, 95% CI = 1.45-1.70), recent (aOR = 1.61, 95% CI = 1.50-1.73), and past (aOR = 1.94, 95% CI = 1.83-2.05) users of atypical antipsychotics had a higher risk of incident pneumonia. Our study revealed that older patients with PD with current, recent, or past use of haloperidol, olanzapine, quetiapine, risperidone, or clozapine had a higher risk of pneumonia, whereas only those currently using chlorpromazine had a higher risk of pneumonia; by contrast, those taking aripiprazole did not have an increased risk of pneumonia.

To the best of our knowledge, ours is the first study to identify the risk factors for pneumonia in those with PD who receive antipsychotic drugs. A univariate Cox proportional hazards analysis indicated that patients with PD with comorbid cerebrovascular disease, congestive heart failure, asthma, COPD, alcoholism, Alzheimer's disease, or epilepsy had a lower risk of incident pneumonia. Our study also revealed that in patients with PD, combined psychiatric disorder (schizophrenia and bipolar disorder) is an independent predictive factor for the development of pneumonia in patients with PD. The risk of pneumococcal disease in each psychiatric group was significantly higher than that for the general population. Patients with psychiatric disorders (e.g., schizophrenia, bipolar disorder, depression, and anxiety) have an increased risk of pneumonia 29. Patients with schizophrenia have an increased risk of pneumonia 30, and all-cause mortality is higher in this group than in the general population 31. This finding is supported by earlier investigations on the *in vitro* production of cytokines in people with these disorders 24. Another study indicated that cerebrovascular disease, congestive heart failure, COPD, and epilepsy are associated with a higher risk of pneumonia in those with PD 32.

Typical antipsychotics act predominantly through dopamine D_2_ receptor antagonism, which also exacerbates parkinsonian motor deficits 33. According to the AGS 2015 Beers criteria, atypical antipsychotics (except for aripiprazole, clozapine, and quetiapine) are unsuitable for patients with PD patients because of the risk of worsening parkinsonian symptoms 34. According to the 2019 Beers criteria, for patients with PD, all antipsychotics have the potential to aggravate parkinsonian symptoms; however, pimavanserin and clozapine appear to be less likely to precipitate worsening of PD. Quetiapine has only been studied in low-quality clinical trials with efficacy reported in five trials and efficacy similar to that of clozapine reported in two others 35. Those with PD treated with quetiapine exhibit increased mortality, with a higher risk of death compared with those not using an antipsychotic; similar results were also observed for olanzapine and risperidone but not for clozapine 36. These data indicate that clozapine may be a suitable medication for individuals with PD who experience psychotic symptoms.

In a study conducted according to the 2015 Beers criteria, Cox regression analyses revealed an increased risk of pneumonia in nursing home residents with PD who were taking inappropriate antipsychotic agents compared with those taking appropriate agents 13. Older people with PD in long-term care who have therapy-related psychosis and use inappropriate antipsychotic medications may experience a deterioration in parkinsonian symptoms 37. Inappropriate antipsychotic medications can potentially affect overall voluntary movements and swallowing movements because of their antagonism of the D_2_ receptors in patients with PD 38, 39. This increased risk of aspiration pneumonia is of particular concern in older patients with PD 40. Our study revealed that clozapine led to the highest risk of pneumonia in older people with PD. This finding is supported by the fact that clozapine has strong anticholinergic effects 18, 19, in addition to inflammatory effects 27.

The limitations of this study are as follows. First, information on some factors affecting pneumonia are unavailable on the LHID, such as alcohol and tobacco consumption behavior and laboratory measurements. There is also no information about the health status and physical performance, such as self-rated health, activities of daily living (ADL), and instrumental activities of daily living (IADL) disability. Furthermore, the LHID only includes information that is part of the health insurance declaration, and medical information from self-funded medical treatment cannot be obtained. Thus, antipsychotic use may be underestimated. Second, the study only used ICD codes to define the disease without consideration of medical procedure codes; this approach may lead to overdiagnosis. Third, some medications that may potentially increase the risk of incident pneumonia were not enrolled in the study, such as proton pump inhibitors (PPIs). In order to focus on the antipsychotics, the study reduced the medication confounding by adjusting comorbidity disease. Besides, NHI has a strict payment guideline for PPIs that are only paid for severe gastrointestinal diseases. Fourth, all participants in the study were above 65 years old. Since older patients are already at higher risk for pneumonia, future studies can establish a younger PD comparison group to verify the effect of antipsychotics on older patients with PD. In addition, the severity of PD and the disease duration of PD may also affect the study results. This study was a nationwide population-based study. Thus, the study results have the accuracy and representativeness. Besides, its observational research design precluded this study from inferring that antipsychotic use causes pneumonia. Future studies should obtain more information from other databases or through questionnaires to infer causality.

In conclusion, older patients with PD receiving typical antipsychotics or atypical antipsychotics had a higher risk of pneumonia. Among these antipsychotics, clozapine had the highest risk of pneumonia. Clinicians should pay attention to the risk of pneumonia in older patients with PD who receive typical antipsychotics and atypical antipsychotics, especially when prescribing antipsychotics with clozapine, to minimize adverse effects.

## Figures and Tables

**Table 1:** Baseline characteristics of older patients with Parkinson's disease

Variables	Pneumonia				p-value
	Without		With		
	N	%	N	%	
Total	40,948	100.00	10,237	100.00	
**Gender**					0.078
Female	21,750	53.12	5,338	52.14	
Male	19,198	46.88	4,899	47.86	
**Age (year) (mean ± SD)**	77.03±5.85		77.10±5.84		0.626
65-70	4,771	11.65	1,174	11.47	
70-75	8,396	20.50	2,109	20.60	
75-80	13,537	33.06	3,368	32.90	
80-85	11,921	29.11	3,037	29.67	
≥85	2,323	5.67	549	5.36	
**Income level**					0.267
Low income (≤21,000)	21,784	53.20	5,476	53.49	
Middle income (21,000-33,000)	11,528	28.15	2,922	28.54	
High income (≥33,000)	7,636	18.65	1,839	17.96	
**Urbanization**					0.622
Urban	27,298	66.67	6,810	66.52	
Suburban	9,042	22.08	2,299	22.46	
Rural	4,608	11.25	1,128	11.02	
**CCI score**					0.827
0	6,977	17.04	1,714	16.74	
1	9,701	23.69	2,457	24.00	
2	9,762	23.84	2,456	23.99	
≥3	14,508	35.43	3,610	35.26	
**Diabetes mellitus**					<0.001
No	25,104	61.31	6,696	65.41	
Yes	15,844	38.69	3,541	34.59	
**Hypertension**					<0.001
No	11,091	27.09	3,207	31.33	
Yes	29,857	72.91	7,030	68.67	
**Cerebrovascular disease**					<0.001
No	19,668	48.03	4,112	40.17	
Yes	21,280	51.97	6,125	59.83	
**Arrhythmia**					0.134
No	33,605	82.07	8,336	81.43	
Yes	7,343	17.93	1,901	18.57	
**Upper respiratory tract infection**					0.944
No	19,948	48.72	4,983	48.68	
Yes	21,000	51.28	5,254	51.32	
**Heart failure**					<0.001
No	34,740	84.84	8,398	82.04	
Yes	6,208	15.16	1,839	17.96	
**Asthma**					<0.001
No	35,733	87.26	7,989	78.04	
Yes	5,215	12.74	2,248	21.96	
**COPD**					<0.001
No	28,002	68.38	4,301	42.01	
Yes	12,946	31.62	5,936	57.99	
**Periodontitis**					0.155
No	40,017	97.73	9,980	97.49	
Yes	931	2.27	257	2.51	
**Chronic kidney disease**					<0.001
No	39,657	96.85	9,980	97.49	
Yes	1,291	3.15	257	2.51	
**Chronic liver disease**					<0.001
No	35,193	85.95	9,141	89.29	
Yes	5,755	14.05	1,096	10.71	
**Alcoholism**					<0.001
No	40,901	99.89	10,202	99.66	
Yes	47	0.11	35	0.34	
**Alzheimer disease**					<0.001
No	36,129	88.23	8,357	81.64	
Yes	4,819	11.77	1,880	18.36	
**Rheumatoid arthritis**					0.078
No	39,789	97.17	9,980	97.49	
Yes	1,159	2.83	257	2.51	
**Cancer**					<0.001
No	33,008	80.61	8,402	82.07	
Yes	7,940	19.39	1,835	17.93	
**Epilepsy**					<0.001
No	39,161	95.64	9,039	88.30	
Yes	1,787	4.36	1,198	11.70	
**Schizophrenia**					<0.001
No	40,263	98.33	9,948	97.18	
Yes	685	1.67	289	2.82	
**Bipolar disorder**					<0.001
No	40,009	97.71	9,869	96.41	
Yes	939	2.29	368	3.59	
**Major depressive disorder**					0.082
No	38,291	93.51	9,524	93.04	
Yes	2,657	6.49	713	6.96	
**Anxiety**					<0.001
No	39,431	96.30	10,047	98.14	
Yes	1,517	3.70	190	1.86	

**Table 2:** Incidence of pneumonia in older patients with Parkinson's disease

Variables	Pneumonia				p-value
	Without		With		
	N	%	N	%	
**Any one of Antipsychotic**					
No	38,030	80.94	8,957	19.06	
Current users	2,918	69.51	1,280	30.49	<0.001
Recent users	3,813	70.12	1,625	29.88	<0.001
Past users	6,959	68.61	3,184	31.39	<0.001
**Typical antipsychotics**					
No	40,595	80.16	10,045	19.84	
Current users	353	64.77	192	35.23	<0.001
Recent users	488	68.83	221	31.17	<0.001
Past users	1,904	71.31	766	28.69	<0.001
**Haloperidol**					
No	40,649	80.14	10,074	19.86	
Current users	299	64.72	163	35.28	<0.001
Recent users	429	69.08	192	30.92	<0.001
Past users	1,658	70.67	688	29.33	<0.001
**Chlorpromazine**					
No	40,891	80.02	10,208	19.98	
Current users	57	66.28	29	33.72	0.002
Recent users	66	68.75	30	31.25	0.006
Past users	299	70.85	123	29.15	<0.001
**Atypical antipsychotics**					
No	38,328	80.78	9,122	19.22	
Current users	2,620	70.15	1,115	29.85	<0.001
Recent users	3,407	70.29	1,440	29.71	<0.001
Past users	5,917	67.72	2,821	32.28	<0.001
**Clozapine**					
No	40,851	80.04	10,185	19.96	
Current users	97	65.10	52	34.90	<0.001
Recent users	122	58.37	87	41.63	<0.001
Past users	245	58.06	177	41.94	<0.001
**Olanzapine**					
No	40,771	80.05	10,160	19.95	
Current users	177	69.69	77	30.31	<0.001
Recent users	236	75.88	75	24.12	0.069
Past users	611	66.41	309	33.59	<0.001
**Quetiapine**					
No	39,128	80.45	9,509	19.55	
Current users	1,820	71.43	728	28.57	<0.001
Recent users	2,391	70.89	982	29.11	<0.001
Past users	4,227	67.48	2,037	32.52	<0.001
**Aripiprazole**					
No	40,878	80.01	10,212	19.99	
Current users	70	73.68	25	26.32	0.124
Recent users	87	75.00	29	25.00	0.178
Past users	255	75.44	83	24.56	0.036
**Risperidone**					
No	40,425	80.23	9,960	19.77	
Current users	523	65.38	277	34.63	<0.001
Recent users	708	67.11	347	32.89	<0.001
Past users	1,990	65.57	1,045	34.43	<0.001

**Table 3:** Association between antipsychotic drugs and pneumonia

Variables	Model 1 ^a^		Model 2 ^b^		Model 3 ^c^	
	OR	95% CI	OR	95% CI	OR	95% CI
**Any one of Antipsychotic**						
No (ref.)	1					
Current users	1.63	1.51-1.75	-	-	-	-
Recent users	1.63	1.52-1.74	-	-	-	-
Past users	1.89	1.80-2.00	-	-	-	-
**Typical antipsychotics**						
No (ref.)			1			
Current users	-	-	1.76	1.46-2.13	-	-
Recent users	-	-	1.46	1.23-1.73	-	-
Past users	-	-	1.10	1.00-1.21	-	-
**Haloperidol**						
No (ref.)					1	
Current users	-	-	-	-	1.72	1.41-2.11
Recent users	-	-	-	-	1.41	1.17-1.69
Past users	-	-	-	-	1.08	0.97-1.19
**Chlorpromazine**						
No (ref.)					1	
Current users	-	-	-	-	1.84	1.13-2.98
Recent users	-	-	-	-	1.44	0.90-2.30
Past users	-	-	-	-	1.13	0.90-1.43
**Atypical antipsychotics**						
No (ref.)			1			
Current users	-	-	1.57	1.45-1.70	-	-
Recent users	-	-	1.61	1.50-1.73	-	-
Past users	-	-	1.94	1.83-2.05	-	-
**Clozapine**						
No (ref.)					1	
Current users	-	-	-	-	1.77	1.24-2.51
Recent users	-	-	-	-	2.60	1.95-3.46
Past users	-	-	-	-	1.81	1.46-2.23
**Olanzapine**						
No (ref.)					1	
Current users	-	-	-	-	1.60	1.21-2.13
Recent users	-	-	-	-	1.15	0.87-1.51
Past users	-	-	-	-	1.20	1.03-1.40
**Quetiapine**						
No (ref.)					1	
Current users	-	-	-	-	1.44	1.31-1.58
Recent users	-	-	-	-	1.53	1.41-1.66
Past users	-	-	-	-	1.71	1.60-1.82
**Aripiprazole**						
No (ref.)					1	
Current users	-	-	-	-	1.56	0.97-2.51
Recent users	-	-	-	-	1.46	0.94-2.26
Past users	-	-	-	-	0.98	0.75-1.27
**Risperidone**						
No (ref.)					1	
Current users	-	-	-	-	1.81	1.55-2.11
Recent users	-	-	-	-	1.62	1.41-1.86
Past users	-	-	-	-	1.52	1.39-1.66
**Comorbidities (Yes vs No)**						
Diabetes mellitus	0.88	0.84-0.92	0.88	0.84-0.92	0.88	0.84-0.92
Hypertension	0.74	0.70-0.78	0.74	0.70-0.78	0.74	0.70-0.78
Cerebrovascular disease	1.19	1.13-1.25	1.19	1.13-1.25	1.19	1.13-1.25
Arrhythmia	0.94	0.89-1.00	0.94	0.89-1.00	0.95	0.89-1.00
Upper respiratory tract infection	0.88	0.84-0.92	0.88	0.84-0.92	0.88	0.84-0.92
Congestive heart failure	1.08	1.01-1.15	1.08	1.01-1.15	1.08	1.01-1.15
Asthma	1.37	1.29-1.46	1.37	1.29-1.46	1.37	1.29-1.46
COPD	2.73	2.60-2.86	2.73	2.60-2.86	2.73	2.60-2.86
Periodontitis	1.02	0.89-1.19	1.02	0.89-1.19	1.02	0.88-1.18
Chronic kidney disease	0.97	0.84-1.11	0.97	0.84-1.11	0.97	0.84-1.11
Chronic liver disease	0.70	0.65-0.75	0.70	0.65-0.75	0.69	0.65-0.75
Alcoholism	1.91	1.22-3.01	1.89	1.20-2.98	1.89	1.19-2.99
Alzheimer disease	1.35	1.27-1.44	1.36	1.27-1.44	1.36	1.28-1.44
Rheumatoid arthritis	0.85	0.74-0.98	0.85	0.74-0.98	0.85	0.74-0.98
Cancer	0.87	0.82-0.92	0.87	0.81-0.92	0.87	0.82-0.92
Epilepsy	2.14	1.98-2.33	2.14	1.98-2.33	2.15	1.98-2.33
Schizophrenia	1.33	1.15-1.54	1.32	1.14-1.54	1.30	1.12-1.51
Bipolar disorder	1.41	1.23-1.61	1.42	1.24-1.62	1.41	1.24-1.62
Major depressive disorder	0.94	0.85-1.03	0.94	0.85-1.03	0.94	0.86-1.03
Anxiety	0.59	0.50-0.69	0.59	0.50-0.69	0.59	0.50-0.69

^a.^ the adjusted model of any one antipsychotic. ^b.^ the adjusted model of typical and atypical antipsychotic. ^c.^ the adjusted model of each individual antipsychotic.

## Abbreviations

PD: Parkinson's disease
AGS: American Geriatrics Society
LHID: longitudinal health insurance database
HWDC: Health and Welfare Data Science Center Taiwan
MOHW: Ministry of Health and Welfare Taiwan
NHI: National Health Insurance
ICD-9-CM: International Classification of Diseases, Ninth Revision, Clinical Modification
ICD-10-CM: International Classification of Diseases, Tenth Revision, Clinical Modification
COPD: chronic obstructive pulmonary disease
aOR: adjusted odds ratios
CI: confidence interval
ADL: activities of daily living
IADL: instrumental activities of daily living
PPIs: proton pump inhibitors
